# Patient Preferences for Chronic Myeloid Leukemia Medication Regimen Attributes and their Potential Impact on Adherence: Results from a Multi-national Conjoint Study

**DOI:** 10.36469/9890

**Published:** 2014-09-03

**Authors:** Ishan Hirji, Jessica Grinspan, Emuella Flood, Diana Chirovsky, Jennifer Devlen, Samuel Wagner, Catherine Davis

**Affiliations:** 1 Bristol-Myers Squibb, Wallingford, CT, USA; 2 ICON PRO, Bethesda, Maryland, USA; 3 Department of Health Policy and Management, Gillings School of Global Public Health University of North Carolina at Chapel Hill, Chapel Hill, NC, USA; 4 Bristol-Myers Squibb, Princeton, NJ, USA

**Keywords:** adherence, regimen, dce, patient preference, cml

## Abstract

**Background:** Tyrosine kinase inhibitors (TKIs) have significantly improved survival for patients with chronic myeloid leukemia (CML) but require long-term administration and non-adherence due to regimen requirements has been reported.

**Objectives:** This study sought to identify how much patients value more convenient regimens and the potential impact that regimen may have on medication adherence.

**Methods:** This cross-sectional, six-country study utilized a web-based discrete conjoint experiment (DCE) survey in which participants selected between hypothetical treatments that differed on three attributes: meal requirements/restrictions, frequency of administration, and monthly co-pay, to quantify willingness to pay. Attribute percent importance ratings were derived from a multinomial logit model, and utilities were summed for each product profile to determine the most preferred regimen profile. Additional survey questions asked about attributes perceived to affect adherence and the ease and convenience of participants’ current regimen.

**Results:** A total of 318 patients completed the survey; median age 53 years (range 18-87); 43.7% male. Four participants were excluded from the conjoint analysis due to illogical responses. The most important regimen attribute driving preferences was the meal requirement/restriction, which was almost twice as important as dose frequency. The majority of participants preferred the profile of a once a day dosing taken with or without a meal, and estimates of willingness-to-pay helped to quantify this preference. In terms of adherence, the majority of participants perceived that having to fast before and after taking medication would be the most likely reason for missing a dose.

**Conclusions:** The results suggest that patients value the convenience of CML treatments and perceive certain regimen characteristics, particularly meal requirements or restrictions, as likely to affect adherence. It is important for healthcare providers to be aware of the potential impact of treatment convenience on non-adherence and communicate closely with patients to decrease this potential.

## BACKGROUND

Chronic Myeloid Leukemia (CML) is characterized by proliferation of myeloid cells in the bone marrow and their accumulation in the blood. Although the annual incidence of 1 to 2 cases per 100,000 population remains constant,[1] therapeutic advances through the use of tyrosine kinase inhibitors (TKIs) have significantly improved survival such that the overall prevalence of CML is expected to continue to increase. In the United States, the number of patients with CML was approximately 70,000 in 2010 and this is estimated to increase to approximately 181,000 in 2050.[2]

As TKIs are typically administered on a long-term basis, and non-adherence may impact clinical outcomes,[3-5] there is a need for better understanding of patient preferences toward specific regimen attributes that can influence therapy choice and adherence.[6] Among the TKIs, in addition to their variable adverse event profile, there are notable differences in dosing schedule, and fasting or meal requirements. Specifically, according to the US Food & Drug Administration (FDA) dosing instructions, imatinib is taken once or twice a day with a meal and large glass of water (Gleevec® FDA). Nilotinib is taken twice a day with water, twelve hours apart, and no food is to be eaten 2 hours before or 1 hour after taking it (Tasigna® FDA). Dasatinib is taken once a day, preferably at the same time, and can be taken with or without food and with or without water (Sprycel® FDA).

Little is known, however, the extent to which patients value more convenient regimens and the impact of regimen on medication adherence. The objective of this study was to understand the value that patients place on more convenient regimens using a willingness-to-pay analysis. A secondary objective was to understand patient perceptions regarding how regimen may influence medication adherence.

## METHODS

This was a cross-sectional web-based survey completed by patients with chronic phase CML (CML-CP) residing in France, Germany, Italy, Spain, United Kingdom and the United States. Eligibility criteria included self-reported diagnosis of CML-CP, current prescription for imatinib, nilotinib or dasatinib, age >18 years, adequate written and oral fluency in language of country. Patients who self-reported being currently in the accelerated or blast phase of CML were excluded. Participants were recruited from February – September 2012 using a specialist market research recruitment agency who used a number of methodologies including recruitment from patient databases, patient associations and through referrals from physicians. The databases contain patients diagnosed with CML who have agreed to be included and to be contacted for research studies. Potential participants listed in the database were contacted by the recruiter via email or telephone with an invitation to participate. Other potential participants were made aware of the study by the medical or nursing staff or through a flyer posted in the health care office, or via notices posted on sites of self-help groups, patient associations or advocacy groups. Interested participants contacted the recruiter and were screened for eligibility and, if willing and eligible, provided with the URL link to the web survey. Upon entering the link, participants were presented with an IRB approved informed consent form. Only those who provided online consent to participate in the study were able to proceed to the online survey. The study protocol was approved by MaGil IRB (Rockville, Maryland).

### Survey Development

To inform the content of the survey questionnaire, qualitative research was conducted among 30 CML-CP patients treated with oral TKIs in France, Germany, Italy, Spain, the United Kingdom and the United States (five per country). In-depth, one-on-one interviews were conducted by a trained, local interviewer in the native language using a standardized moderator guide that included open-ended questions and probes asking about instructions for taking their CML medication, difficulties in taking medication as prescribed, preferences for regimen characteristics and adherence. Interviews were audio-recorded, transcribed and coded using thematic analysis[7], assisted by qualitative data analysis software, MaxQDA version 10 (Berlin, Germany).

A draft survey, comprising three sections, was designed. One section was a conjoint survey eliciting preferences for regimen attributes, discussed in more detail below. A second section included questions regarding participants' experiences with their current CML medication, in which they were asked to rate how easy or difficult and how convenient or inconvenient it was to take their current medication as prescribed. Ratings were made on an 11-point numerical rating scale (NRS) where 0=very difficult/very inconvenient to 10=very easy/very convenient. Additionally, participants were asked to choose the most preferred regimen profile among four that matched the four regimens being evaluated. They also were asked questions on adherence, factors influencing their adherence to their current CML medication and to choose which treatment attribute if any, would be the most likely cause for them to miss a dose or take a dose later than scheduled. A final section included socio-demographic and clinical background questions. The survey was programmed using Sawtooth Software (SSI Web version 7), hosted on a secure server and took approximately 20 minutes to complete. The draft survey underwent an iterative process of pilot-testing with a total of 16 patients (2-3 patients in each country), who completed the survey online and participated in an interview to provide feedback. Based on the results of the pilot testing, the wording of the following two attributes was changed to improve clarity: "you take it with or without a meal" was changed to "you have the option to take it with or without a meal" and "you take it with a large glass of water and a meal" was changed to "you take it with a meal and a large glass of water". Other revisions included adding questions for twice-daily dosing for imatinib in addition to the once-daily dosing already included, adding response options for the reasons for non-adherence to fully capture patient-reported experiences, and changes to the translation of specific words to more accurately match the intended meaning in the English language version.

Conjoint analysis, also referred to as discrete-choice experiments, is a useful and widely used method to quantify preferences and has been increasingly utilized in health care studies to explore patient preferences for treatment attributes.[8-9] Study participants are presented with hypothetical scenarios comprising two or more alternative treatment options and are asked to make a choice between the treatments, requiring a trade-off between attributes.[10] The relative importance of these attributes is thus determined by participants' stated choices over the entire set of tasks.

Selection of treatment regimen attributes and levels per attribute for the conjoint analysis section of the questionnaire was based on the regimen requirements of the three target medications, i.e. dosing schedule and fasting/meal requirement.[11] To assess the value that patients place on specific treatment regimen attributes, out-of-pocket cost was also included as an attribute in the conjoint survey, which allowed for a willingness-to-pay analysis. The attributes and levels were: a) meal requirements or restrictions (with a meal and a large glass of water; with water but cannot eat 2 hours before and 1 hour after; option to take with or without a meal and with or without water); b) frequency (number and timing of doses per day: once a day at the same time every day; twice a day, 12 hours apart) and c) out-of-pocket cost ($0; $25; $50 [or local equivalent]) for 1 month's worth of the medication. The cost levels were selected to quantify the value patients place on regimen attributes, not to reflect real-world co-pays, particularly as these vary considerably by country, and were informed by patient interviews aimed at gauging cost sensitivity.

The conjoint survey included 13 choice tasks, in which participants were asked to choose between two hypothetical medications, each with a different full regimen profile comprised of the three attributes (meal requirements/restrictions, frequency, and cost; as depicted in Figure 1). Twelve choice tasks were presented based on an orthogonal design,[12] and, to identify participants who might not understand the task, one of the choice tasks (presented midway through the survey) was designed to include a clear winner; that is, one product profile was superior over the other (more convenient and less costly than the alternative).

**Figure 1.** Screen Shot of Sample Conjoint Choice Task

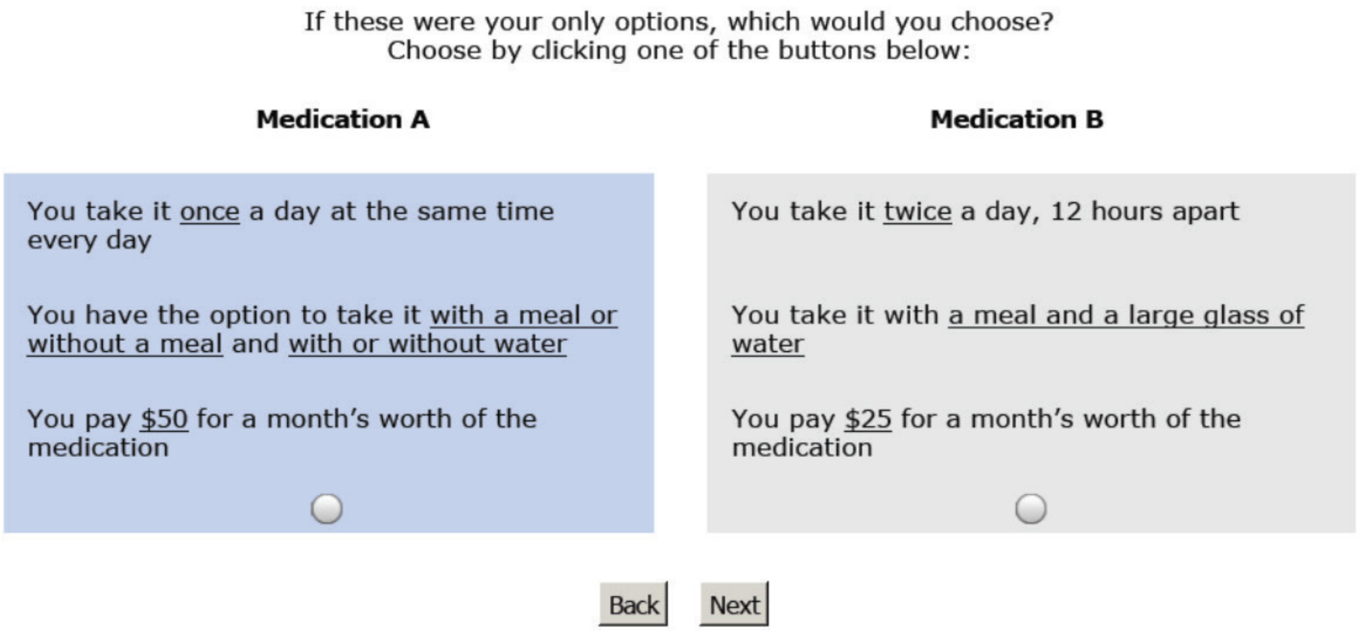

## Analysis

The conjoint data were analyzed using Sawtooth Software (Sequim, Washington) v7.0.26, the CBC/Hierarchical Bayes (HB) module, and the Sawtooth Market Simulator. Preference weights were calculated using a multinomial logit model. Logit analysis is an iterative procedure to find the maximum likelihood solution for fitting a multinomial logit model to the data.[13] The HB approach was applied to the data to further refine the precision of the utility estimates.[13] HB produces more robust parameter estimates than the logit model alone by "borrowing" information from the overall sample (means and covariances) to further refine the preference weights of respondents in the dataset.[14-16]

The parameter estimates from conjoint analysis enable the calculation of the relative importance of each attribute. Specifically, the relative importance was calculated at the respondent-level by dividing the range of each attribute (utility of highest level- utility of lowest level) by the sum of ranges of all attributes, and multiplying it by 100.[17] These estimates indicate how much the difference in importance between the best and worst levels of each attribute affects the decision to choose a treatment. These are ratio data. For example, an attribute with an importance of 10% is twice as important as an attribute with an importance of 5%.

Regimen profiles were created from the attribute levels to match the product labels for the four medication scenarios: imatinib once a day, imatinib twice a day, nilotinib and dasatinib, and a series of analyses were performed examining patient preferences for these derived profiles. Market simulator software was used to create a base case in which the regimen profiles would compete against each other for market share of preference. It is important to note that these results do not reflect actual market share for these medications, as this study focused solely on preferences for regimen attributes, and did not consider other characteristics such as efficacy and side effects that are likely to influence patients' decision to choose a specific treatment profile. Using the out-of-pocket cost levels, incremental willingness to pay amounts for moving among attribute levels were estimated through a series of sensitivity analyses. For instance, sensitivity analyses would estimate the amount patients would be willing to pay per month for a change from a product with a specified treatment regimen attribute, for example a meal requirement, to another product with a different regimen attribute, for example, the option to take it with or without a meal (no meal requirement).

SAS 9.0 was used for statistical analysis of the remaining survey questions and socio-demographic and clinical data. Descriptive analyses, including frequency and percentages for categorical data, and means and standard deviations for continuous data, were conducted. Significance testing was performed using chi-square for categorical variables or analysis of variance (ANOVA) for continuous variables.

## RESULTS

A total of 326 patients were screened and eligible, of which 318 completed the survey comprising the final sample. Four participants were excluded from the conjoint analysis because they responded incorrectly to the 'clear winner' question. The median age of the sample was 53 years; 43.7% were male, and the median time since CML diagnosis was 3.9 years (Table 1). Approximately one-half (N=177) of participants were currently taking imatinib, of whom 88% were on a once-a-day regimen and 12% were on a twice-a-day regimen; 21% were taking nilotinib, and 23% were taking dasatinib. Five percent of the sample had switched from another CML treatment to their current medication. No significant differences were observed among countries or among medication subgroups with respect to all demographic and clinical characteristics (all $p>0.05$).

### Relative Importance of Attributes

Figure 2 shows the relative importance of the three attributes by country. In the overall sample, meal requirement/restriction (relative importance = 39.0%) and monthly cost (relative importance = 38.5%) were almost twice as important as administration frequency (relative importance = 22.6%) Similar findings were observed in Germany, the United Kingdom, and the United States. In contrast, in France and Italy, meal requirement/restriction was more than twice as important and two-thirds more important than monthly cost, and, in Spain, cost was almost three times more important than meal requirement/restriction (Figure 2). Relative importance estimates were comparable among age and time since diagnosis subgroups.

### Preference Weights for Each Attribute Level

Table 2 shows the preference weights for each attribute level from the conjoint analysis. Within each attribute, the highest preference weight identifies the most favorable level and the lowest weight the least favorable level. The actual values are arbitrary; however, the magnitude of the differences in weights within each attribute is important. For example, the difference in preference between taking medication with a meal and a large glass of water and taking while fasting (difference of 90.2) is more than four times the difference in preference between having the option to take it with/without meal or water and taking it with a meal and a large glass of water (difference of 20.6 i.e. 110.8 minus 90.2).

**Table 1.** Socio-demographic and Clinical Characteristics

| | | Current Medication+ | | | |
|---|---|---|---|---|---|
| | Total Sample (N=318) | Imatinib once a day (n=156) | Imatinib twice a day (n=21) | Nilotinib (n=67) | Dasatinib (n=74) |
| **Age (years)** | | | | | |
| Mean (SD) | 52.3 (12.5) | 52.1 (13.1) | 50.3 (13.3) | 52.8 (11.0) | 52.7 (12.4) |
| Median [range] | 53.0 [18-87] | 51.0 [20-85] | 50.0 [18-79] | 54.0 [28-74] | 55.0 [19-87] |
| **Sex [n (%)]** | | | | | |
| Male | 139 (43.7) | 73 (46.8) | 9 (42.9) | 29 (43.3) | 28 (37.8) |
| **Race [n (%)]** | | | | | |
| White | 309 (97.2) | 153 (98.1) | 20 (95.2) | 66 (98.5) | 70 (94.6) |
| Black | 2 (0.6) | 1 (0.6) | . | . | 1 (1.4) |
| Asian | 1 (0.3) | 1 (0.6) | . | . | . |
| Other | 6 (1.9) | 1 (0.6) | 1 (4.8) | 1 (1.5) | 3 (4.1) |
| **Education* [n (%)]** | | | | | |
| Less than high school | 50 (15.7) | 27 (17.3) | 5 (23.8) | 15 (22.4) | 3 (4.1) |
| High school | 89 (28.0) | 41 (26.3) | 5 (23.8) | 15 (22.4) | 28 (37.8) |
| Some college | 69 (21.7) | 34 (21.8) | 5 (23.8) | 11 (16.4) | 19 (25.7) |
| College degree | 70 (22.0) | 36 (23.1) | 4 (19.1) | 16 (23.9) | 14 (18.9) |
| Graduate degree | 40 (12.6) | 18 (11.5) | 2 (9.5) | 10 (14.9) | 10 (13.5) |
| **Employment [n (%)]** | | | | | |
| Full time | 114 (35.9) | 56 (35.9) | 9 (42.9) | 23 (34.3) | 26 (35.1) |
| Part time | 40 (12.6) | 17 (10.9) | 4 (19.1) | 11 (16.4) | 8 (10.8) |
| Retired | 65 (20.4) | 37 (23.7) | 3 (14.3) | 9 (13.4) | 16 (21.6) |
| Student | 6 (1.9) | 2 (1.3) | 1 (4.8) | 1 (1.5) | 2 (2.7) |
| Unemployed | 9 (2.8) | 6 (3.9) | . | 3 (4.5) | . |
| Homemaker | 31 (9.8) | 18 (11.5) | 2 (9.5) | 5 (7.5) | 6 (8.1) |
| Temporarily disabled | 8 (2.5) | 1 (0.6) | 1 (4.8) | 4 (6.0) | 2 (2.7) |
| Permanently disabled | 29 (9.1) | 12 (7.7) | 1 (4.8) | 7 (10.5) | 9 (12.2) |
| Other | 16 (5.0) | 7 (4.5) | . | 4 (6.0) | 5 (6.8) |
| **Years since diagnosis** | | | | | |
| Mean (SD) | 5.1 (4.6) | 5.6 (4.6) | 3.7 (3.7) | 4.5 (4.3) | 5.0 (4.7) |
| Median [range] | 3.9 [1–24] | 4.2 [1-24] | 2.3 [1-13] | 3.4 [1-23] | 3.0 [1-20] |

*Or equivalent terms in other countries; +No significant differences were observed across countries or medication subgroups for any of the sociodemographic or clinical variables (all p>0.05).

### Willingness to Pay

Based on the preference weights for each attribute level, it was estimated that participants would be willing to pay approximately $22 (or local equivalent) to be able to have the option to take their medication with or without a meal instead of having to fast (Table 3). Participants in France would pay the most of all countries (equivalent of $40), and those in Spain would pay the least (equivalent of $4.25) for this option. In contrast, participants, as a whole, were willing to pay less than $1 (or local equivalent) to be able to have the option to take their medication with or without a meal instead of taking their medication with a meal and a large glass of water. However, this finding differed substantially among countries in which participants in France and Italy placed relatively low importance on cost; these participants would pay $24.50 and $8, respectively, to be able to have the option to take their medication with or without a meal over the meal requirement. No substantial differences were observed among groups differing by age. Participants diagnosed more than 2 years ago were willing to pay more than those diagnosed less than 2 years ago to change from a fasting requirement to an option to take with or without a meal ($30 vs. $12, not tabulated).

**Figure 2.** Relative Importance* Estimates for each Attribute by Country

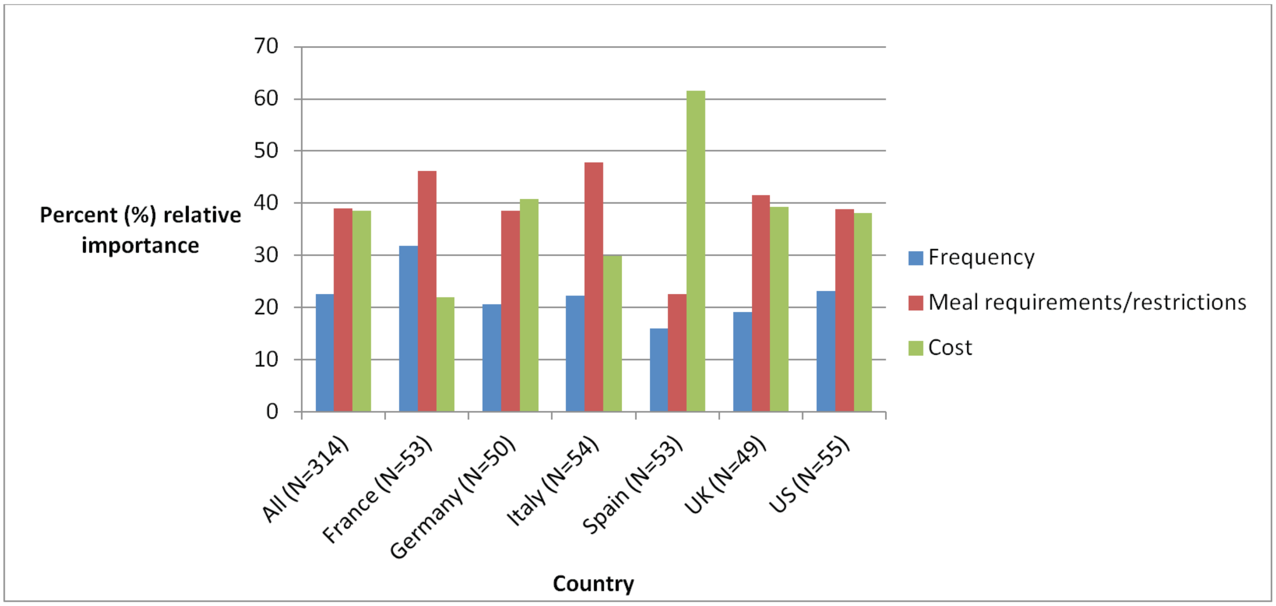

*Relative importance percentages are ratio data: 40% is twice as important as 20%

**Table 2.** Mean Preference Weights for each Attribute Level

| Attribute and Respective Levels | Preference Weight | Standard Error |
|---|---|---|
| **Frequency** | | |
| Once a day at the same time | 62.8 | 1.6 |
| Twice a day, 12 hours apart | 0.0 | 1.6 |
| **Meal Requirements/Restrictions** | | |
| Option with/without meal/water | 110.8 | 1.7 |
| With a meal and large glass of water | 90.2 | 1.5 |
| With water but fasting | 0.0 | 2.4 |
| **Cost per month*** | | |
| $0 | 108.6 | 2.6 |
| $25 | 57.9 | 0.4 |
| $50 | 0.0 | 2.6 |

*Or equivalent in local currency: (UK: £0, £15 £30; France, Germany, Italy and Spain: €0, €20, €40)

*Patient Preferences for CML Medication Profiles*

Based on the preference estimates for the regimen profiles derived from attribute level preference weights, 65%, 24%, 8%, and 3% of participants were estimated to prefer dasatinib, imatinib (once a day), imatinib (twice a day), and nilotinib, respectively; preference estimates did not vary by participants' current medication (p >0.05). These estimated proportions did not vary substantially across groups differing by country, current medication, treatment history (i.e., having experience with only one versus more than one medication), age, and time since diagnosis. These estimated proportions derived from preference weights were consistent with the responses identifying which of the four regimens were preferred by the sample; the percentages choosing profiles matching to dasatinib, imatinib (once a day), imatinib (twice a day), and nilotinib regimens were 62%, 29%, 7%, and 2%, respectively.

**Table 3.** Estimated Willingness to Pay for the option to take Medication with or without a Meal, Stratified by Country

| Original Attribute Level | All (N=314) | France (N=53) | Germany (N=50) | Italy (N=54) | Spain (N=53) | UK (N=49) | US (N=55) |
|---|---|---|---|---|---|---|---|
| From fasting requirement (nilotinib regimen) | $22 | $40 | $24 | $20 | $4.25 | $18 | $13 |
| From meal requirement (imatinib regimen) | <$1 | $24.50 | <$1 | $8 | <$1 | <$1 | <$1 |

*Ease and Convenience and Adherence to Treatment Regimen*

When asked to rate the ease and convenience of their current treatment regimen, participants taking dasatinib had the highest mean scores for both ease and convenience, whereas participants taking nilotinib rated their regimen as the most difficult and inconvenient (Figure 3). When asked, hypothetically, which treatment attribute, if any, would most likely cause them to miss a dose of medication, fasting twice a day, 12 hours apart was selected by 46% of the sample; whereas taking medication twice a day, 12 hours apart was selected by 9% (Table 4). Thirty-six percent of participants reported that hypothetically, none of the regimen attributes would cause them to miss a dose. There were significant differences across the CML treatment groups ($p<0.001$). Participants currently taking nilotinib were more likely to report that regimen attributes would have no influence on missing doses (60% for nilotinib participants vs. 36% for the total sample). Although fasting was the most commonly reported reason for potentially missing a dose across all treatment groups, it was least commonly cited as a reason to miss for those on nilotinib.

**Table 4.** Participants' Perceptions of the most Likely Cause for Missing Doses by Current Medication[*]

| | Current Medication | | | |
|---|---|---|---|---|
| Most likely cause for missing doses | All (N=318) | Imatinib (N=177) | Nilotinib (N=67) | Dasatinib (N=74) |
| Having to take it once a day at the same time each day | 10 (3%) | 8 (5%) | 2 (3%) | 0 (0%) |
| Having to take it twice a day, twelve hours apart | 30 (9%) | 14 (8%) | 5 (7%) | 11 (15%) |
| Having to take it with a meal | 15 (5%) | 12 (7%) | 2 (3%) | 1 (1%) |
| Having to take it with a large glass of water | 2 (0.6%) | 0 (0%) | 0 (0%) | 2 (3%) |
| Having to take it with water | 1 (0.3%) | 1 (1%) | 0 (0%) | 0 (0%) |
| Having to fast (not eat) 2 hours before and 1 hour after taking it, twice a day, 12 hours apart | 145 (46%) | 81 (46%) | 18 (27%) | 46 (62%) |
| None of the above – would never miss a dose | 115 (36%) | 61 (35%) | 40 (60%) | 14 (19%) |

[*]$p<0.001$ across medication subgroups for each reason

**Figure 3.** Ease and Convenience of Current Treatment Regimen*,+

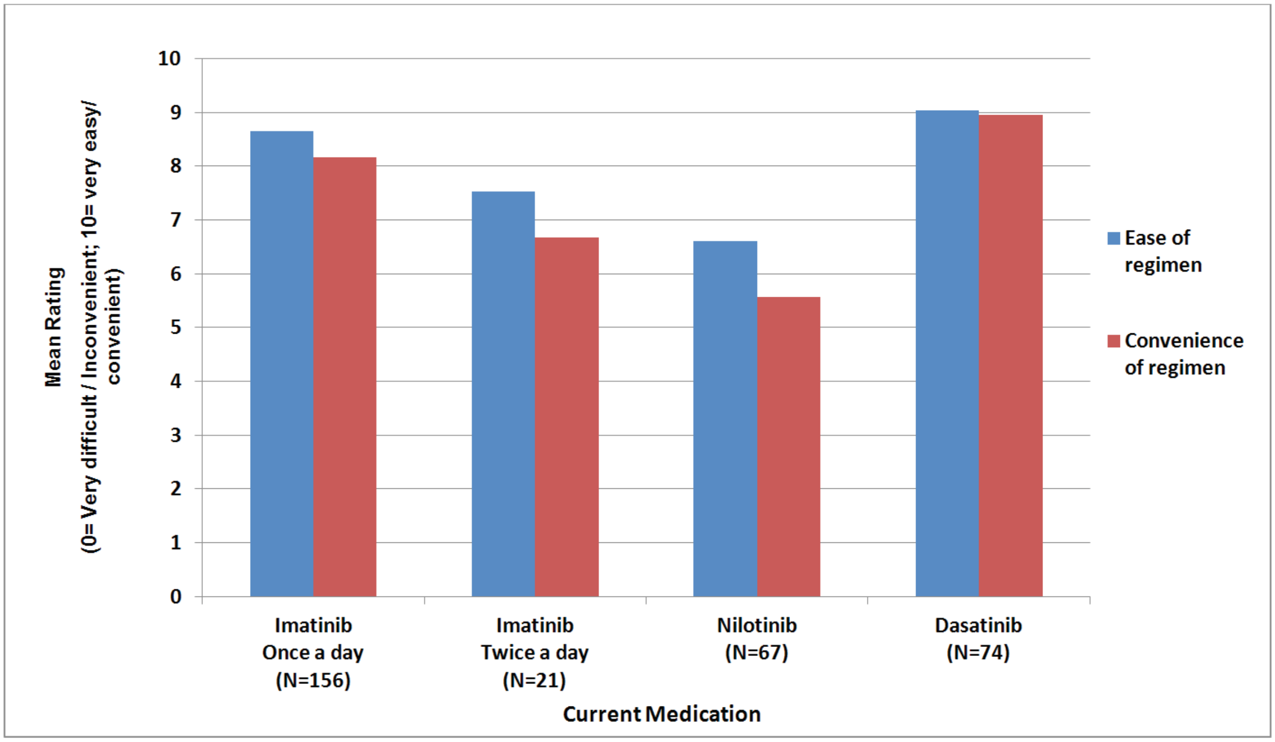

*ANOVA p<0.001; +Ease of regimen refers to how "easy or difficult" patients perceived their regimen to be; convenience of regimen refers to how "convenient or inconvenient" they perceived their regimen to be.

## DISCUSSION

Oral TKIs have substantially improved the management of CML-CP, but current TKI medication regimens differ in frequency of doses per day and food requirements/restrictions. As a result, increased attention is needed to better understand the importance of different aspects of CML treatment regimen and their potential impact on adherence. The results of this study suggest that patients with CML-CP value treatment convenience, particularly not having to fast.

Conjoint analysis proved to be a useful method to quantify patient preferences for treatment regimen attributes. Only a very small number of study participants did not understand the task, and results from a separate survey question asking participants to choose the most preferred regimen supported the conjoint findings. While the finding that participants most prefer the option to take medication with or without a meal and least prefer having to fast was not surprising, the conjoint analyses helped to quantify these preferences. Participants were willing to pay to have the option to take their medication with or without a meal rather than having to fast. On the other hand, participants' willingness to pay to have the option to take medication with or without a meal over the meal requirement was relatively small and, in some countries, negligible. Feedback from patient interviews to support the development of the survey questionnaire revealed that, in practice, many patients take their medication with a meal partly because theroutine acts as a reminder, thus increasing adherence. However, in the conjoint survey, participants clearly preferred to have the option to take their medication with or without a meal. Patient interviews suggested that even if patients generally took their medication with food, there were occasions, such as when travelling, when it might be difficult to access sufficient food to meet the requirement of a meal. Thus, this option would allow them the freedom to take their medication as scheduled, potentially increasing adherence. Adherence to medication is critical to achieving a good outcome with CML, and even modest levels of non-adherence have been reported to be associated with poorer outcomes.[3,5] Therefore, minimizing missed or late doses is important in the management of CML. In the current study, when asked hypothetically, all participants, regardless of their current regimen, reported that a fasting requirement would be the most likely reason for non-adherence. It was noteworthy that nilotinib participants were the group most likely to report that they would never miss a dose due to regimen characteristics, suggesting that they tolerate the strict regimen. In contrast, few participants identified two doses per day as an important determinant of non-adherence. Further research, using direct measurements of adherence, is needed to better understand whether these patient perceptions do in fact impact non-adherence behaviors.

While most of the findings were consistent across all countries, there were some differences to note. Cost was a more important attribute for participants in Spain than in most other countries. Differences in health care and reimbursement systems or the economic environment may be possible explanations, but exploring possible reasons was beyond the scope of this project and could form the basis of future research.

The clinical experts interviewed to help inform the development of the survey suggested that patient preferences and dislikes for treatment regimens may be influenced by patient characteristics, such as age or time since diagnosis. This study sought to explore these characteristics; however, subgroup analyses suggested that preferences were generally consistent across age and year since diagnosis.

To our knowledge, this study is the first to provide a comprehensive analysis of patient preferences for different regimen attributes of CML treatment and their potential impact on treatment adherence. Nevertheless, the study has limitations. Treatment attributes of efficacy and side effects are likely to have a considerably greater influence than regimen on patient preferences for treatment. However, our qualitative research suggested that regimen is also an important attribute and may influence adherence. In order to be able to explore the relative importance of regimen attributes and the value of convenience, the current study only focused on dosing schedules and fasting or meal requirements and excluded efficacy and side effects. Thus, the results regarding preferences for treatment need to be interpreted with this in mind. Perceptions of adherence obtained in this study are subject to bias. It is interesting to note that participants currently taking nilotinib were the least likely of all treatment groups to perceive fasting as a cause for missing a dose. The current study is not able to answer whether these patients were taking nilotinib because of their ability to adhere to this particular regimen, or whether patients underestimate their ability to adapt, and those currently on other treatments might be more adherent than they perceive if required to switch. In any case, a regimen which requires dose administration while fasting (more than once a day) is perceived to be most challenging by all. Despite efforts to obtain a diverse sample with respect to socio-demographic and clinical characteristics, those who participated in the survey may have been younger and more computer literate than the general population of CML patients, which may affect the generalizability of the results. Additionally, patients who elected to be included in a database for participation in research studies, or patients who responded to an invitation or notice about the current study may not be representative of the CML population. Generalizability may also be limited given that, while there is a slight male preponderance within the general population of CML patients (male to female ratio of 1.4:1)[18], only 43.7% of participants were male within our sample.

## CONCLUSIONS

The results of the present study suggest that, when efficacy and safety are not taken in consideration, patients value the convenience of CML treatments and perceive certain regimens as likely to affect adherence. In particular, patients who currently have to fast rate their treatment regimen as more difficult and less convenient than those who do not. Furthermore, regardless of patients' current medication or fasting requirements, the attribute of fasting was perceived as one of the most likely reasons for patient-reported non-adherence. Therefore, it is important for healthcare providers to be aware of the potential impact of treatment convenience on non-adherence and communicate closely with patients to decrease this potential. Further studies investigating how these factors are valued by patients when elements of treatment response and tolerability to therapy are included in the equation will be important.

## DECLARATION OF COMPETING INTERESTS

The research presented in this manuscript was funded entirely by Bristol-Myers Squibb. Bristol-Myers Squibb is also the funding body for manuscript preparation and submission. IH, SW and CD were employees of Bristol-Myers Squibb at the time this study was conducted. DC is a graduate student at University of North Carolina, Chapel Hill and is the recipient of a pre-doctoral fellowship from Bristol-Myers Squibb. Oxford Outcomes, Inc., an ICON plc company, employed EF, JD, and JG at the time this study was conducted. Oxford Outcomes was contracted by Bristol-Myers Squibb to conduct this study and prepare the manuscript. There are no other conflicts of interest.
